# CXCL14 Deficiency in Mice Attenuates Obesity and Inhibits Feeding Behavior in a Novel Environment

**DOI:** 10.1371/journal.pone.0010321

**Published:** 2010-04-23

**Authors:** Kosuke Tanegashima, Shiki Okamoto, Yuki Nakayama, Choji Taya, Hiroshi Shitara, Rie Ishii, Hiromichi Yonekawa, Yasuhiko Minokoshi, Takahiko Hara

**Affiliations:** 1 Stem Cell Project group, The Tokyo Metropolitan Institute of Medical Science, Tokyo Metropolitan Organization for Medical Research, Kamikitazawa, Setagaya-ku, Tokyo, Japan; 2 Division of Endocrinology and Metabolism, National Institute for Physiological Sciences, Nishigonaka Myodaiji, Okazaki, Aichi, Japan; 3 Priority Organization for Innovation and Excellence, Kumamoto University, Kurokami, Kumamoto-shi, Kumamoto, Japan; 4 Laboratory of Mouse Model for Human Heritable Diseases, The Tokyo Metropolitan Institute of Medical Science, Tokyo Metropolitan Organization for Medical Research, Kamikitazawa, Setagaya-ku, Tokyo, Japan; University of Camerino, Italy

## Abstract

**Background:**

CXCL14 is a chemoattractant for macrophages and immature dendritic cells. We recently reported that *CXCL14*-deficient (*CXCL14* ^−/−^) female mice in the mixed background are protected from obesity-induced hyperglycemia and insulin resistance. The decreased macrophage infiltration into visceral adipose tissues and the increased insulin sensitivity of skeletal muscle contributed to these phenotypes.

**Methodology/Principal Findings:**

In this study, we performed a comprehensive study for the body weight control of *CXCL14* ^−/−^ mice in the C57BL/6 background. We show that both male and female *CXCL14* ^−/−^ mice have a 7–11% lower body weight compared to *CXCL14* ^+/−^ and *CXCL14* ^+/+^ mice in adulthood. This is mainly caused by decreased food intake, and not by increased energy expenditure or locomotor activity. Reduced body weight resulting from the CXCL14 deficiency was more pronounced in double mutant CXCL14^−/−^ *ob*/*ob* and *CXCL14* ^−/−^A^y^ mice. In the case of *CXCL14* ^−/−^A^y^ mice, oxygen consumption was increased compared to *CXCL14* ^+/−^A^y^ mice, in addition to the reduced food intake. In *CXCL14* ^−/−^ mice, fasting-induced up-regulation of *Npy* and *Agrp* mRNAs in the hypothalamus was blunted. As intracerebroventricular injection of recombinant CXCL14 did not change the food intake of *CXCL14* ^−/−^ mice, CXCL14 could indirectly regulate appetite. Intriguingly, the food intake of *CXCL14* ^−/−^ mice was significantly repressed when mice were transferred to a novel environment.

**Conclusions/Significance:**

We demonstrated that CXCL14 is involved in the body weight control leading to the fully obese phenotype in leptin-deficient or A^y^ mutant mice. In addition, we obtained evidence indicating that CXCL14 may play an important role in central nervous system regulation of feeding behavior.

## Introduction

Obesity is caused by increased caloric intake and decreased energy expenditure. Many secretory peptides and hormones are involved in the metabolic pathways that regulate feeding behavior and energy homeostasis. The hypothalamus and brainstem integrate satiety and hunger signals elicited by peripheral regulators, such as leptin and centrally produced orexigenic molecules such as neuropeptide Y (NPY) and agouti-related protein (AgRP) [1], [2].

CXCL14 (also known as BRAK) is a member of the CXC chemokine family [3], [4]. CXCL14 exhibits chemotactic activity for macrophages, dendritic precursor cells, and natural killer cells. A CXCL14 receptor has not yet been identified. Although CXCL14-deficient (*CXCL14* ^−/−^) mice do not display severe defects in their immune systems [5], we recently found that *CXCL14* ^−/−^ female mice weigh significantly less than wild-type mice and are protected from obesity-induced hyperglycemia, hyperinsulinemia, hypoadiponectinemia, and insulin resistance [6]. Expression of *CXCL14* is upregulated in adipose tissue and skeletal muscle in obese mice. Detailed characterization of high fat diet-fed *CXCL14* ^−/−^ female mice revealed that CXCL14 regulates glucose metabolism in two ways: through recruitment of macrophages to visceral white adipose tissue, and through partial inhibition of insulin signaling pathways in skeletal muscle. It is of note that the blood insulin levels of *CXCL14* ^−/−^ female mice are lower than those of control female mice irrespective of obesity. However, the molecular basis of the gender-specific phenotype of CXCL14^−/−^ mice has not yet been determined.

Although *CXCL14* is abundantly expressed in the brain, its physiological role remains unclear. Major metabolic regulators such as leptin and adiponectin are known to play important roles in both skeletal muscle and the hypothalamus in the regulation of energy metabolism. Therefore, we hypothesized that CXCL14 might also play a role in the control of appetite and/or energy expenditure through the central nervous system. In this study, we present evidence that CXCL14 indirectly regulates food intake and is required for body weight gain in two genetic mouse models of obesity, *ob*/*ob* and A^y^ mice. In addition, we demonstrate that CXCL14-deficient mice take a longer time to adapt to a new environment before initiating feeding behavior.

## Results

### Characterization of *CXCL14* ^−/−^ mice

We have previously reported that female *CXCL14* ^−/−^ mice have lower body weight than *CXCL14* ^+/−^ mice on a C57BL/6-CBA mixed background [6]. To elucidate the cause of this body weight phenotype, we backcrossed *CXCL14* ^+/−^ mice with C57BL/6 mice for more than 10 generations. Unexpectedly, the birth rate of *CXCL14* ^−/−^ mice from heterozygous pairs was approximately half of the expected Mendelian ratio for both male and female offspring (Table 1). *CXCL14* ^−/−^ male mice were fertile. In contrast, *CXCL14* ^−/−^ female mice became pregnant, but in most cases they failed to deliver or nurse the newborn pups (Table 2).

**Table 1:** Birth rate and fertility of *CXCL14* ^−/−^ mice.

	Genotypes of F1 mice from (+/−) male x (+/−) female crosses			
	(+/+)	(+/−)	(−/−)	Total
Male	31	54	12 (12.4%*)	97
Female	27	35	10 (13.9%*)	72
Total	58	89	22 (13%*)	169

	Genotypes of F1 mice from (−/−) male x (+/−) female crosses			
		(+/−)	(−/−)	Total
Male		115	44 (27.7%**)	159
Female		89	37 (29.4%**)	126
Total		204	81 (28.4%**)	285

	Parturition of pregnant (−/−) females			
	Failure	Dead pups	Success	Total
	4	9	2	15

Expected birth ratios are 25% (*) and 50% (**), respectively.

**Table 2:** Primers for RT-PCR analysis used in this study.

Gene	Forward primer (5′-3′)	Reverse primer (5′-3′)	Size (bp)
*CXCL14*	CCAAGATTCGCTATAGCGAC	CCTGCGCTTCTCGTTCCAGG	191
*Npy*	CTCCGCTCTGCGACACTACA	AATCAGTGTCTCAGGGCTGGA	75
*Agrp*	GCGGAGGTGCTAGATCCACA	AGGACTCGTGCAGCCTTACAC	69
*Pomc*	ACCTCACCACGGAGAGCAAC	GCGAGAGGTCGAGTTTGCA	59
*Socs3*	CCTTTGACAAGCGGACTCTC	GCCAGCATAAAAACCCTTCA	215
*Cart*	CGAGAAGAAGTACGGCCAAG	GGAATATGGGAACCGAAGGT	132
*GAPDH*	AAATTCAACGGCACAGTCAA	GAACGGACGGAGATGATGAC	218

*CXCL14* ^−/−^ mice were not easily bred, even by crossing *CXCL14* ^+/−^ dams with *CXCL14* ^−/−^ male mice (Table 1). Since *CXCL14* ^−/−^ mice were successfully produced by *in vitro* fertilization of *CXCL14* ^−/−^ eggs with *CXCL14* ^−/−^ sperm, the lower birth rate of *CXCL14* ^−/−^ mice could be caused by perinatal selection rather than developmental defects. In fact, there was a tendency for single litters from *CXCL14* ^−/−^ males x *CXCL14* ^+/−^ dams to either have zero or more than 3 null pups. Thus, *CXCL14* ^−/−^ pups might be weaker than heterozygous pups and may be out-competed by their littermates.

### *CXCL14* ^−/−^ mice exhibit lower body weights

**Figure 1:**
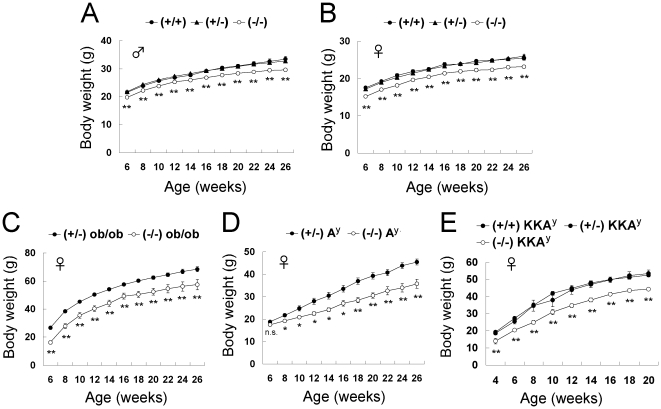
Lower body weights in *CXCL14* ^−/−^ mice and *CXCL14* ^−/−^ crosses with obese mutant mice. Body weight change of male *CXCL14* ^+/+^ (*n* = 12), *CXCL14* ^+/−^ (*n* = 76) and *CXCL14* ^−/−^ mice (*n* = 37) (A); female *CXCL14* ^+/+^ (*n* = 13), *CXCL14* ^+/−^ (*n* = 38) and *CXCL14* ^−/−^ (*n* = 19) mice (B); female *CXCL14* ^+/−^ *ob*/*ob* (*n* = 9) and *CXCL14* ^−/−^ *ob*/*ob* (*n* = 4) mice (C); female *CXCL14* ^+/−^A^y^ (*n* = 11) and *CXCL14* ^−/−^A^y^ (*n* = 5) mice (D); and female *CXCL14* ^+/+^KKA^y^ (*n* = 4), *CXCL14* ^+/−^KKA^y^ (*n* = 13) and *CXCL14* ^−/−^KKA^y^ mice (*n* = 5) (E), are shown. Each data point represents the mean ± SEM. *, *P*<0.05; **, *P*<0.01; n.s., not significant compared to the corresponding value for *CXCL14* ^+/−^, *CXCL14* ^+/−^ *ob*/*ob*, *CXCL14* ^+/−^A^y^, or *CXCL14* ^+/−^KKA^y^ control mice.

We compared the growth curves of *CXCL14* ^−/−^ mice with those of *CXCL14* ^+/−^ and *CXCL14* ^+/+^ mice in the C57BL/6 background between 6 weeks and 26 weeks of age. Among both males and females, the body weights of *CXCL14* ^−/−^ mice were 7–11% lower than those of their heterozygous and wild-type littermates (Figure 1A, B). Mean body weight of 3-week-old female *CXCL14* ^−/−^ mice (6.6±0.26 g) was significantly lower than that of female *CXCL14* ^+/−^ littermates (7.9±0.34 g; *n* = 5, *P* = 0.0479), indicating that the lighter body weight of *CXCL14* ^−/−^ mice emerges at the weaning period. As the metabolism-related phenotypes of *CXCL14* ^−/−^ mice were more prominent in females in initial experiments, subsequent experiments were carried out using female *CXCL14* ^−/−^ mice and *CXCL14* ^+/−^ littermates.

We next generated double mutant mice by crossing *CXCL14* ^−/−^ and leptin-deficient *ob*/*ob* mice (*CXCL14* ^−/−^ *ob*/*ob*), and compared their growth with *CXCL14* ^+/−^ *ob*/*ob* mice. CXCL14 deficiency resulted in a 14–39% lower body weight in *ob/ob* mice (Figure 1C). This was also the case when we crossed *CXCL14* ^−/−^ with another obese mutant mouse model, A^y^, in which agouti protein is ectopically overexpressed in the hypothalamus. *CXCL14* ^−/−^A^y^ mice were significantly lighter than *CXCL14* ^+/−^A^y^ mice (Figure 1D). Moreover, this finding was reproduced in KKA^y^ mice, a more hyperphagic A^y^ mouse strain (Figure 1E). Taken together, these data show that the lower body weight resulting from CXCL14 deficiency moderates obesity caused by genetic hyperphagia and reduced energy expenditure. We thus presume that the point of action of CXCL14 is genetically downstream or independent of the *ob*/*ob* and A^y^ mutations.

**Figure 2:**
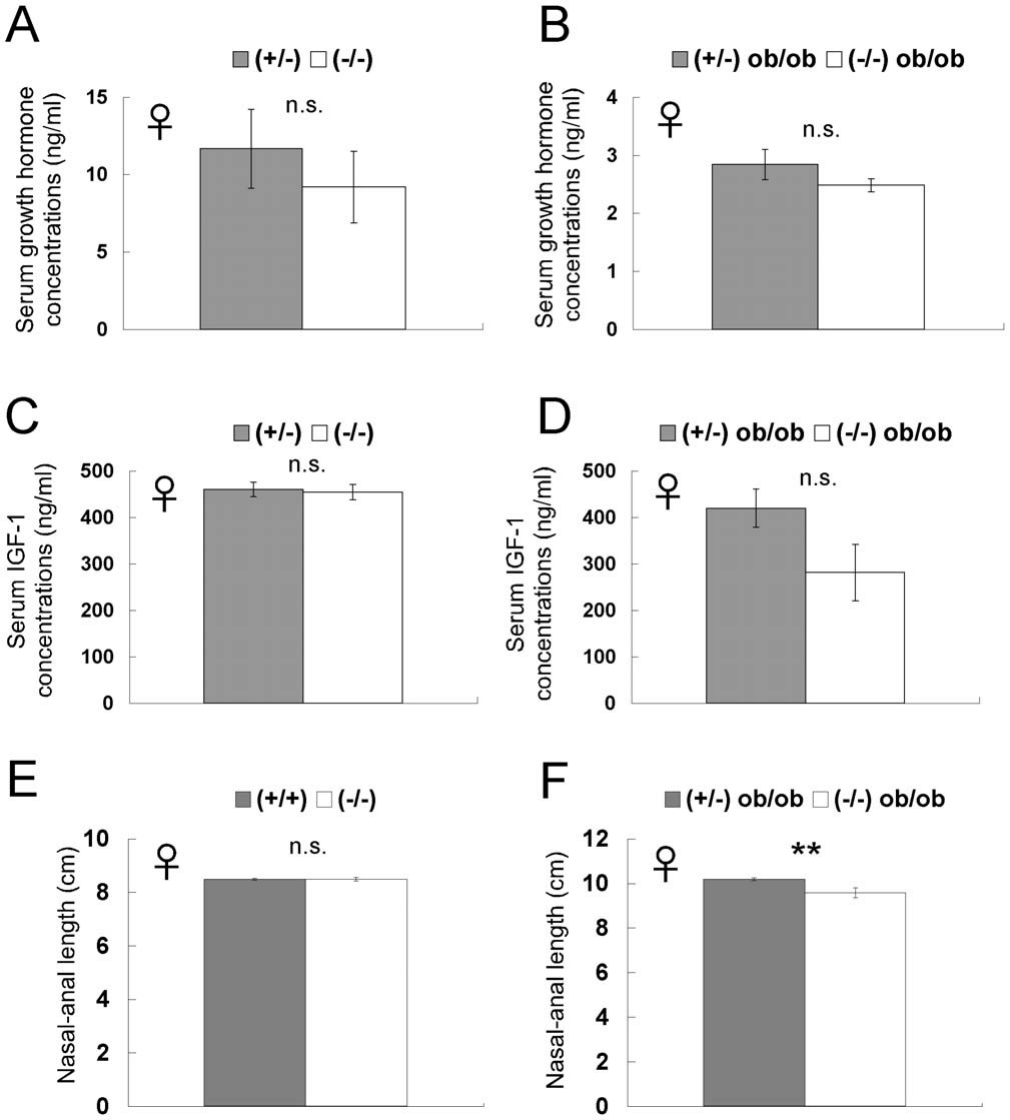
Comparison of growth hormone/IGF-1 levels and nose-anus lengths between *CXCL14* ^+/−^ and *CXCL14* ^−/−^ female mice. (A, B) Daytime concentrations of serum growth hormone in 6-week-old female *CXCL14* ^+/−^ (*n* = 10) and *CXCL14* ^−/−^ mice (*n* = 9) (A), and 12-week-old female *CXCL14* ^+/−^ *ob*/*ob* (*n* = 4) and *CXCL14* ^−/−^ *ob*/*ob* (*n* = 4) mice (B). (C, D) Daytime concentrations of serum IGF-1 in 6-week-old female *CXCL14* ^+/−^ (*n* = 8) and *CXCL14* ^−/−^mice (*n* = 8) (C), and 12-week-old female *CXCL14* ^+/−^ *ob*/*ob* (*n* = 4) and *CXCL14* ^−/−^ *ob*/*ob* (*n* = 4) mice (D). (E, F) The mean nasal-anal lengths of 15-week-old female *CXCL14* ^+/+^ (*n* = 6) and *CXCL14* ^−/−^mice (*n* = 6) (E), and 6-month-old female *CXCL14* ^+/−^ *ob*/*ob* (*n* = 4) and *CXCL14* ^−/−^ *ob*/*ob* (*n* = 10) mice (F). Each data point represents the mean ± SEM. **, *P*<0.01; n.s., not significant compared to the corresponding value for *CXCL14* ^+/−^ or *CXCL14* ^+/+^ control mice.

To examine whether growth retardation contributes to the body weight phenotype of *CXCL14* ^−/−^ mice, we compared concentrations of growth hormone and IGF-I in the circulation of *CXCL14* ^−/−^ and *CXCL14* ^+/−^ mice, and of *CXCL14* ^−/−^ *ob*/*ob* and *CXCL14* ^+/−^ *ob*/*ob* mice. We found no significant difference in either comparison (Figure 2A–D). As previously reported [7], reduced growth hormone levels associated with the *ob*/*ob* mutation were observed in *CXCL14* ^−/−^ *ob*/*ob* mice when they were compared to *CXCL14* ^−/−^ mice (Figure 2A, B). However, the serum growth hormone concentrations of *CXCL14* ^−/−^ *ob*/*ob* mice were indistinguishable from those of *CXCL14* ^+/−^ *ob*/*ob* mice, ruling out the involvement of the growth hormone/IGF-I axis in the lighter body weight phenotype of *CXCL14* ^−/−^ mice. Mean nose-anus length of *CXCL14* ^−/−^ mice was nearly identical to that of *CXCL14* ^+/−^ mice (Figure 2E). In contrast, however, mean nasal-anal length of *CXCL14* ^−/−^ *ob*/*ob* mice was significantly shorter than *CXCL14* ^+/−^ *ob*/*ob* mice (Figure 2F). As we have previously reported the shorter nose-anus length of *CXCL14* ^−/−^ mice fed a high fat diet [6], CXCL14 could be involved in the body length regulation under the obese conditions.

### Reduced food intake is a major cause of lower body weight in *CXCL14* ^−/−^ mice

**Figure 3:**
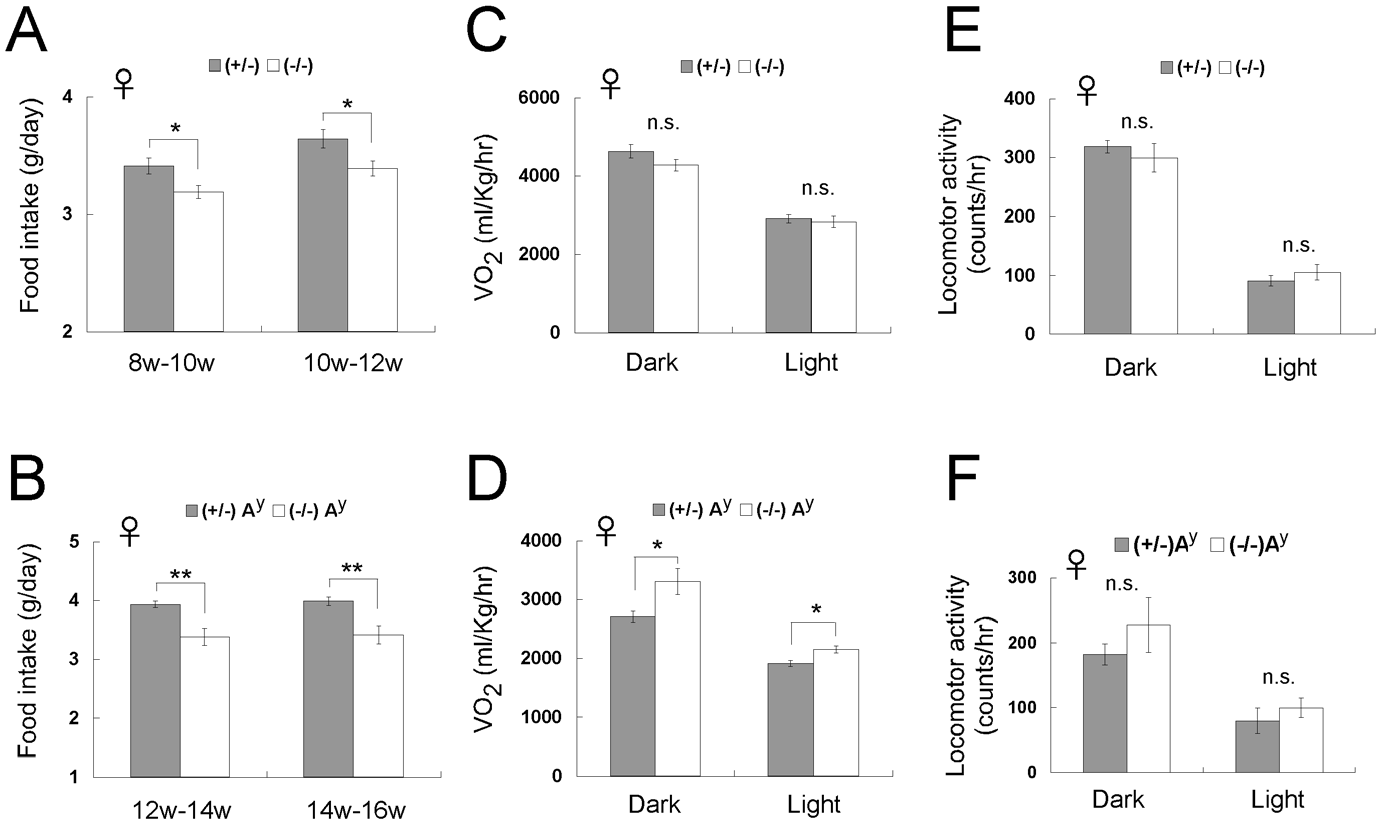
Comparison of food intake, energy expenditure, and locomotor activity between *CXCL14* ^+/−^ and *CXCL14* ^−/−^ female mice. (A, B) Daily food intake of female *CXCL14* ^+/−^ (*n* = 8) and *CXCL14* ^−/−^ (*n* = 7) mice (A), and female *CXCL14* ^+/−^A^y^ (*n* = 8) and *CXCL14* ^−/−^A^y^ (*n* = 4) mice (B). (C–F) Oxygen consumption of female *CXCL14* ^+/−^ (*n* = 6) and *CXCL14* ^−/−^ (*n* = 6) mice (C), and female *CXCL14* ^+/−^A^y^ (*n* = 6) and *CXCL14* ^−/−^A^y^ (*n* = 3) mice (D), and locomotor activity of female *CXCL14* ^+/−^ (*n* = 4) and *CXCL14* ^−/−^ (*n* = 6) mice (E), and female *CXCL14* ^+/−^A^y^ (*n* = 8) and *CXCL14* ^−/−^A^y^ (*n* = 5) mice (F) during night- and daytime are shown. Sixteen to 20 week-old mice were used for these experiments. Each data point represents the mean ± SEM. *, *P*<0.05; **, *P*<0.01; n.s., not significant compared to the corresponding value for *CXCL14* ^+/−^ control mice.

**Figure 4:**
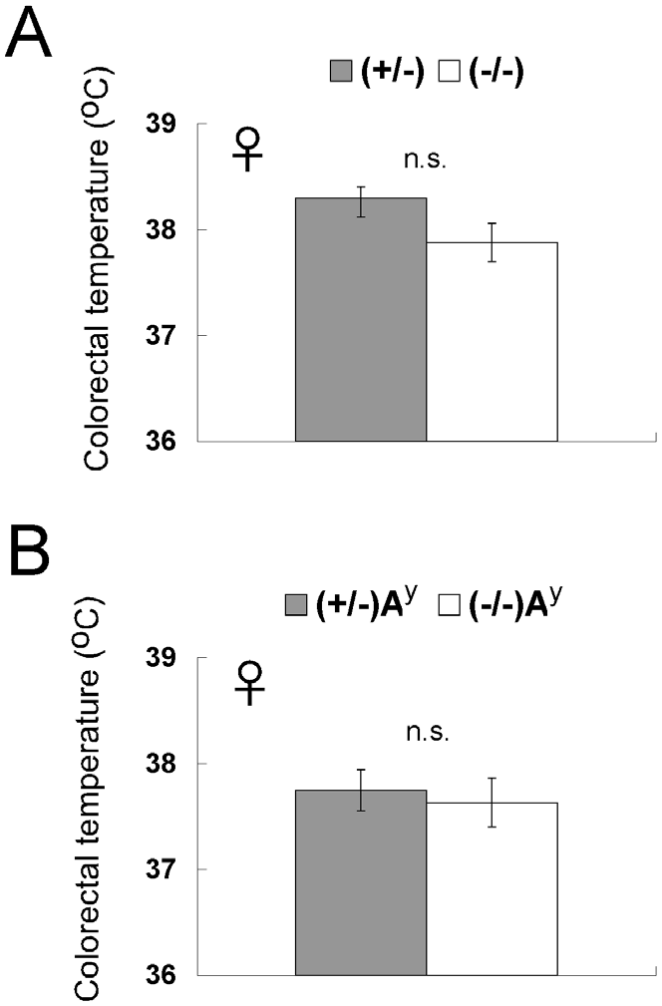
Body temperature is not affected by CXCL14 deficiency. Core body temperatures of female *CXCL14* ^+/−^ (*n* = 6) and *CXCL14* ^−/−^ (*n* = 6) mice (A), and female *CXCL14* ^+/−^A^y^ (*n* = 6) and *CXCL14* ^−/−^A^y^ (*n* = 3) mice (B). Eighteen to 22 week-old mice were used. Each data point represents the mean ± SEM. n.s., not significant compared to the corresponding value for *CXCL14* ^+/−^ control mice.

To determine whether the lower body weight of *CXCL14* ^−/−^ mice was due to reduced food intake or increased energy expenditure, we measured the daily food intake of *CXCL14* ^+/−^ and *CXCL14* ^−/−^ mice from 8–12 weeks of age, and *CXCL14* ^+/−^A^y^ and *CXCL14* ^−/−^A^y^ mice from 12–16 weeks of age. *CXCL14* ^−/−^ mice ate 6–7% less food than *CXCL14* ^+/−^ mice (Fig. 3A), whereas *CXCL14* ^−/−^A^y^ mice ate 14% less than *CXCL14* ^+/−^A^y^ mice (Figure 3B). However, oxygen consumption and locomotor activity were not significantly different between *CXCL14* ^+/−^ and *CXCL14* ^−/−^ mice either at night or during the daytime (Figure 3C, E). In *CXCL14* ^−/−^A^y^ mice, oxygen consumption was increased compared to *CXCL14* ^+/−^A^y^ mice (Figure 3D). We did not detect any significant difference in locomotor activity between *CXCL14* ^+/−^A^y^ and *CXCL14* ^−/−^A^y^ mice (Figure 3F). These data suggest that a reduction in food intake is a major cause of the lower body weight of *CXCL14* ^−/−^ mice. In addition, enhanced energy expenditure could contribute in part to the lower body weight of *CXCL14* ^−/−^A^y^ mice. Core body temperature was not significantly changed by CXCL14 deficiency in either normal or A^y^ mice (Figure 4A, B).

### Expression of appetite regulators in *CXCL14*-deficient mice

**Figure 5:**
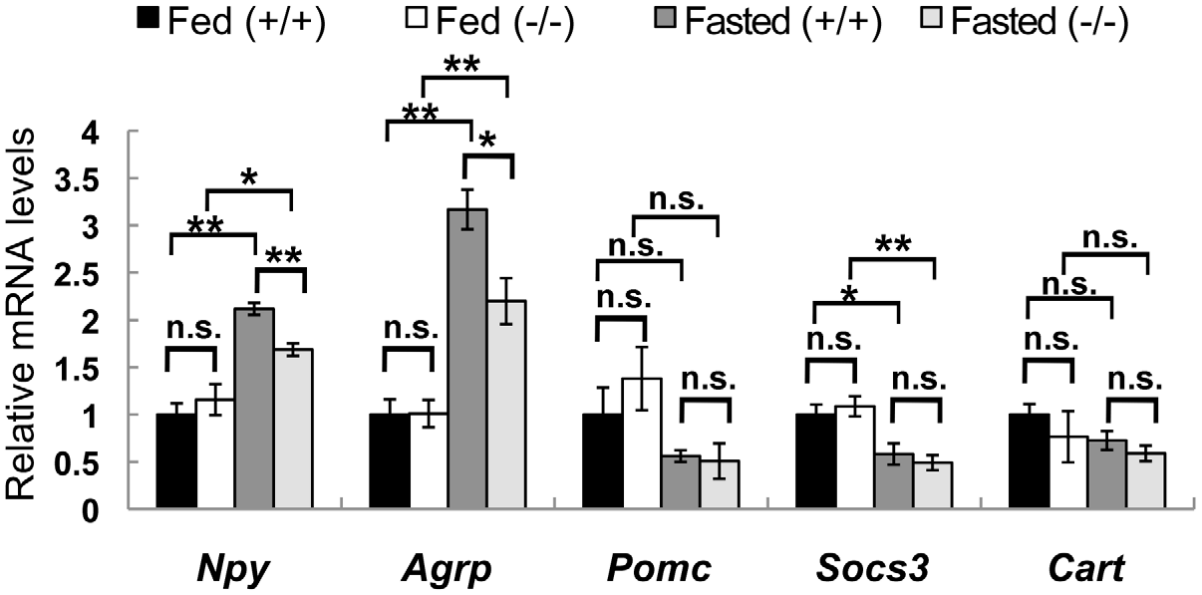
CXCL14 deficiency attenuates fasting-induced up-regulation of *Npy* and *Agrp* mRNAs in the hypothalamus. Relative mRNA expression levels of appetite-regulating molecules (*Npy*, *Agrp*, *Pomc*, *Socs3* and *Cart*) in the hypothalami of female *CXCL14* ^+/+^ and *CXCL14* ^−/−^ mice. Eighteen to 22 week-old mice were used. *GAPDH* was used as the normalization control. Each data point represents the mean ± SEM (*n* = 3). *, *P*<0.05; **, *P*<0.01; n.s., not significant compared to the corresponding value for *CXCL14* ^+/+^ control mice.

We next examined mRNA expression of appetite-regulating peptides in the hypothalamus using real-time RT-PCR. Under the *ad libitum* condition of a standard diet, expression levels of *Npy*, *Agrp*, *Proopiomelanocortin* (*Pomc*), *Socs3*, and *Cocaine and amphetamine-regulated transcript* (*Cart*) were comparable between *CXCL14* ^+/+^ and *CXCL14* ^−/−^ mice (Figure 5), and between *CXCL14* ^+/−^A^y^ and *CXCL14* ^−/−^A^y^ mice (data not shown). However, fasting-induced up-regulation of *Npy* and *Agrp* mRNAs was observed, but significantly blunted in the hypothalami of *CXCL14* ^−/−^ mice compared to control *CXCL14* ^+/+^ mice (Figure 5). In contrast, mRNA level of *Socs3* was lowered in the hypothalami of fasted *CXCL14* ^−/−^ mice as similar as fasted control mice (Figure 5), implying that a critical downstream regulator of the leptin-mediated anorexigenic signaling pathway is not severely affected in *CXCL14* ^−/−^ mice. There was no difference in the expressions of *Pomc* and *Cart* between two groups under the fasted condition (Figure 5).

### Effect of intracerebroventricular (ICV) or intraperitoneal (IP) administration of CXCL14 to the food intake of *CXCL14* ^−/−^ mice

**Figure 6:**
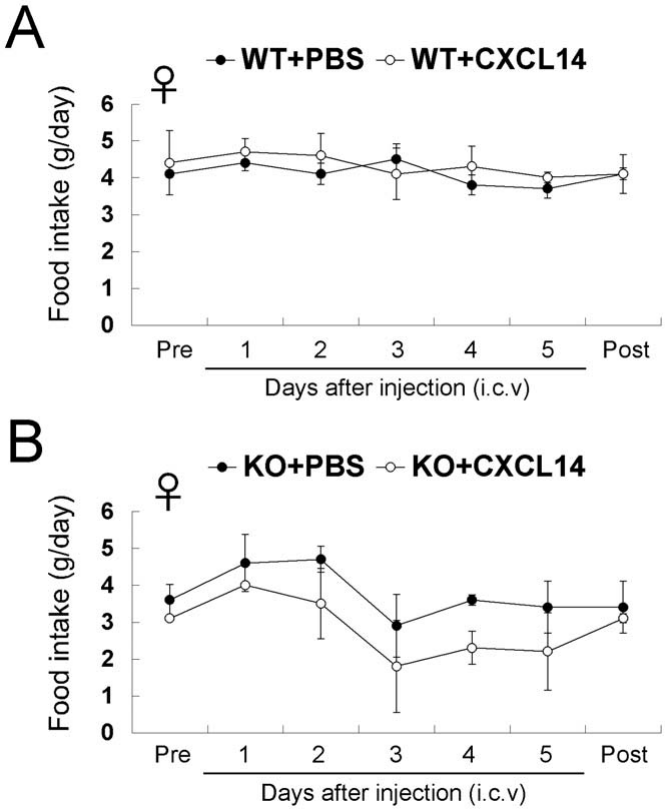
Effects of ICV injection of CXCL14 protein in *CXCL14* ^+/+^ and *CXCL14* ^−/−^ mice. Female *CXCL14* ^+/+^ mice (WT, *n* = 6) (A) and *CXCL14* ^−/−^ mice (KO, *n* = 5) (B) were subjected to ICV injection of recombinant CXCL14 or PBS. Daily food intake measurements before and after the ICV injection in a single experiment are separately shown. Sixteen to 20 week-old mice were used for this experiment. Each data point represents the mean ± SEM. None of the data points for CXCL14-injected samples were statistically significant from the corresponding values for the PBS injection control.

To determine whether CXCL14 acts directly on appetite-regulating neurons, we first injected recombinant CXCL14 into the cerebroventricular cavity of *CXCL14* ^−/−^ mice. The biological activity of CXCL14 was verified using a chemotaxis assay (data not shown). We used age- and sex-matched *CXCL14* ^+/+^ mice as controls. This experiment was made more difficult by the fact that *CXCL14* ^−/−^ mice showed severe anorexia after the first ICV injection, regardless of whether PBS or CXCL14 was injected. This phenomenon was not observed in *CXCL14* ^+/+^ mice. After a 3-week interval, we performed a second and third set of experiments in which *CXCL14* ^−/−^ mice were adapted to this experimental procedure such that they returned to normal feeding. In both *CXCL14* ^+/+^ and *CXCL14* ^−/−^ mice, ICV injection of CXCL14 did not significantly increase food intake (Figure 6A, B).

**Figure 7:**
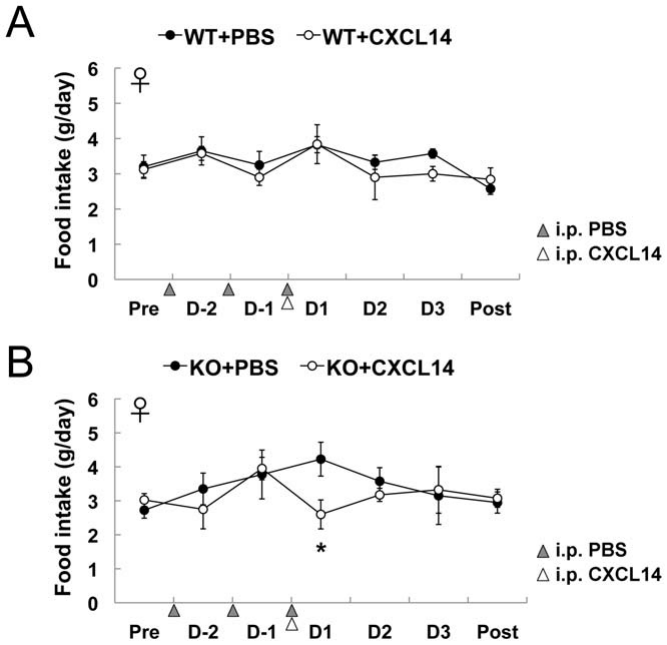
Effects of IP injection of CXCL14 protein in *CXCL14* ^+/+^ and *CXCL14* ^−/−^ mice. Female *CXCL14* ^+/+^ mice (WT, *n* = 4) (A) and *CXCL14* ^−/−^ mice (KO, *n* = 4) (B) were intraperitoneally injected with recombinant CXCL14 or PBS. To habituate mice to the IP injection, all mice were intraperitoneally injected with PBS at indicated time points (shaded arrowheads) before CXCL14 injection (open arrowheads). Daily food intake measurements before and after injection in a single experiment are separately shown. Twelve to 14 week-old mice were used for this experiment. Each data point represents the mean ± SEM. *, *P*<0.05 compared to the PBS control. Except for one point, all other data points were statistically insignificant from the corresponding values for the PBS injection controls.

Next we performed IP injection of recombinant CXCL14 into *CXCL14* ^−/−^ mice to understand the systemic effect of CXCL14. Mice were injected with PBS for two days before CXCL14 administration to be habituated to the procedure. IP injection of CXCL14 into control *CXCL14* ^+/+^ mice did not significantly affect their food intake (Figure 7A). Unexpectedly, however, *CXCL14* ^−/−^ mice ate a lesser amount of food during a day just after the IP injection of CXCL14 when compared to PBS-injected controls (Figure 7B). This effect was transient, as food intake of CXCL14-injected *CXCL14* ^−/−^ mice was returned to normal levels on the second day. Serum concentrations of CXCL14 in age/sex-matched mice injected with CXCL14 (2 µg/g body weight) were 90 ng/ml in average (*n* = 3) at 1 hour after injection and became under the detection level (<2.5 ng/ml) at 3 hours after injection, indicating a rapid degradation of recombinant CXCL14 *in vivo*.

### The feeding behavior of *CXCL14* ^−/−^ mice is sensitive to environmental changes

**Figure 8:**
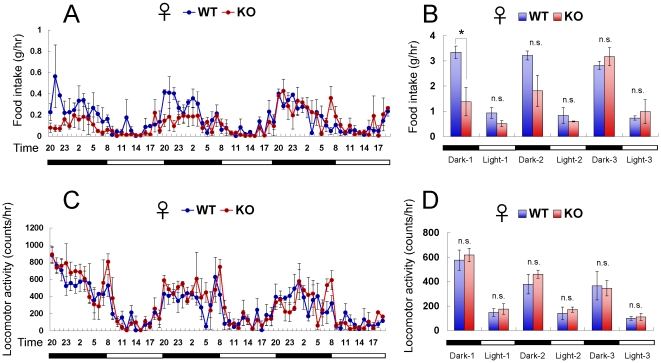
Suppressed feeding behavior of *CXCL14* ^−/−^ mice in a novel environment. Female mice were transferred to the locomotor activity monitoring cage without habituation. Food intake (A, B) and locomotor activity (C, D) of *CXCL14* ^+/+^ mice (WT, *n* = 3) and *CXCL14* ^−/−^ mice (KO, *n* = 3) during the 3 subsequent night-day cycles are shown. Sixteen to 20 week-old mice were used for this experiment. Each data point represents the mean ± SEM. *, *P*<0.05; n.s., not significant compared to the corresponding value for *CXCL14* ^+/+^ control mice.

To obtain further insights into the feeding behavior of *CXCL14* ^−/−^ mice, we individually transferred *CXCL14* ^+/+^ and *CXCL14* ^−/−^ mice from their regular home cages to a locomotor behavior monitoring cage without any habituation. The food intake of *CXCL14* ^−/−^ mice was severely repressed during the first night when compared to wild-type mice, but it gradually recovered to normal levels over the next 3 days (Figure 8A, B). In contrast, locomotor activity was not significantly different between non-habituated *CXCL14* ^+/+^ and *CXCL14* ^−/−^ mice (Figure 8C, D). These data clearly indicate that CXCL14 is required for normal adaptation to a novel environment and initiation of feeding behavior. As the food intake of *CXCL14* ^−/−^ mice in their home cages was constant even after transferring them to fresh cages, the lower body weights seen in *CXCL14* ^−/−^ mice were not due to delayed adaptation to routine cage changes.

## Discussion

Although CXCL14 is known to be a chemoattractant for macrophages and immature dendritic cells, CXCL14-deficient mice exhibit unexpected phenotypes: lower body weight and a reduced birth rate. In this study, we explored the underlying mechanisms of the former phenotype. A major cause of the lower body weight of *CXCL14* ^−/−^ mice is lower food intake, but not increased energy expenditure. Since weaned *CXCL14* ^−/−^ mice were already lighter than *CXCL14* ^+/−^ littermates, reduced capacity of food intake in *CXCL14* ^−/−^ mice should be established during the postnatal three weeks, thereby gaining body weight in proportion to keep the slightly lighter phenotype over the life time. The lighter body weight phenotype was more prominent in two representative hyperphagic mutant mouse strains, *ob*/*ob* and A^y^. The daily food intake of *CXCL14* ^−/−^A^y^ mice was approximately 86% of that of *CXCL14* ^+/−^A^y^ mice. In addition, the basal energy expenditure of *CXCL14* ^−/−^A^y^ mice was significantly higher than that of *CXCL14* ^+/−^A^y^ mice. These two properties protected *CXCL14* ^−/−^A^y^ mice from being obese. It is known that in AgRP/NPY neurons in the hypothalamus, agouti protein antagonizes melanocortin-4 receptors [8], which act to decrease appetite and increase energy expenditure. Intriguingly, we found that decreased expression of *Npy* and *Agrp* mRNAs in the hypothalami of fasted *CXCL14* ^−/−^ mice when compared to fasted control mice. Hence, in *CXCL14* ^−/−^ mice, a signal transduction pathway for the fasting-induced up-regulation of *Npy/Agrp* genes could be partially impaired. Alternatively, it is possible that AgRP/NPY neurons themselves are reduced in *CXCL14* ^−/−^ mice. Further investigations are necessary to distinguish above possibilities and uncover the roles of CXCL14 in the hypothalamus.

The ICV injection of CXCL14 did not stimulate food intake in *CXCL14* ^+/+^ mice or *CXCL14* ^−/−^ mice, suggesting that CXCL14 is not a typical orexigenic hormone. However, the finding of attenuated induction of *Npy* and *Agrp* mRNAs in the hypothalami of fasted *CXCL14* ^−/−^ mice supports the idea that CXCL14 modulates the expression of appetite-regulators. At this moment, we do not know the reason why food intake of *CXCL14* ^−/−^ mice was repressed by the IP injection of CXCL14. Since CXCL14 is inhibitory for the insulin-mediated glucose uptake in skeletal muscle and the serum insulin concentrations of *CXCL14* ^−/−^ mice are lower than those of *CXCL14* ^+/−^ mice [6], temporal increase of blood glucose levels might contribute to the observed phenomenon.

In this study, we revealed the possibility that *CXCL14* ^−/−^ mice are more sensitive to novelty-induced stress. Feeding behavior can be indirectly affected by neuronal abnormalities. In the mouse brain, *CXCL14* mRNA is most abundantly expressed in the cortex, hippocampus and cerebellum (http://www.brain-map.org provided by the Allen Institute for Brain Science). Among appetite-related regions, paraventricular hypothalamus, suprachiasmatic nucleus and piriform cortex show a relatively stronger expression of *CXCL14* mRNA. Expression levels of *CXCL14* in the arcuate nucleus and ventromedial nucleus of the hypothalamus are low. We confirmed the expression of *CXCL14* mRNA in the cortex, hippocampus and hypothalamus of adult mice by RT-PCR (YN, SO, YM, TH, unpublished data). As *CXCL14* is present not only in the hypothalamus, but also in the cortex and hippocampus, we speculate that CXCL14 may be required for the establishment of neural circuits that are closely linked with feeding behavior. Consistent with this, the feeding behavior of *CXCL14* ^−/−^ mice was repressed on the first night after they were transferred to a novel environment. However, the locomotor activity of the mutant mice was not significantly different during this time; thus, it is unlikely that the novelty feeding-suppression phenotype of *CXCL14* ^−/−^ mice is a result of fear. This trait may be related to impairment of anti-depressive functions in the brain. It has been previously demonstrated that novelty-suppressed feeding is directly associated with serotonergic neurons in the dentate gyrus [9], which are established during the first 3 weeks after birth [10]. We plan to subject *CXCL14* ^−/−^ mice to a pathological diagnosis of the brain as well as a battery of behavioral tests, including the novelty-suppressed feeding test.

We have previously reported that female, but not male, *CXCL14* ^−/−^ mice show amelioration of obesity-induced insulin resistance [6]. In female *CXCL14* ^−/−^ mice on the C57BL/6 genetic background, serum insulin concentrations were significantly lower than those of *CXCL14* ^+/−^ female mice (TH, unpublished data). This phenotype was not observed in male *CXCL14* ^−/−^ mice. In contrast, in the present study, we show that the reduced birth rate and lower body weight of *CXCL14* ^−/−^ mice are seen in both males and females. During the ICV injection experiments, we noticed that female *CXCL14* ^−/−^ mice were not as aggressive as wild-type mice. This tendency was also observed in male *CXCL14* ^−/−^ mice. Therefore, the behavioral differences seen in *CXCL14* ^−/−^ mice are not gender-specific, which is in sharp contrast to the metabolic phenotypes of *CXCL14* ^−/−^ mice.

Among CC and CXC chemokine family members, only CXCL14 and CXCL12 are well conserved from fish to humans [11]. Only two amino acid residues of CXCL14 are different between mice and humans. Both of these chemokines are abundantly expressed in the brain. CXCL12 has been shown to be essential for neurogenesis in the fetus through recruitment of neuronal precursor cells [12]–[14]. In this study, we have provided the first evidence for neuronal defects in CXCL14-deficient mice. They do not appear to be global, but rather more specifically related to novelty-associated feeding behavior. Considering the fact that the peripheral functions of CXCL14 are pro-diabetic, CXCL14 may be an important metabolic regulator for the maintenance of energy stores. As CXCL14 may function in multiple regions of the brain, it is important to determine how its functions in various brain regions are coordinated to regulate feeding behavior.

Finally, here we presented evidence demonstrating that disruption of *CXCL14* results in reduced body weight using two representative genetic mouse models of obesity. *CXCL14*-deficiency resulted in repression of feeding behavior in a novel environment without affecting locomotor activity. Therefore, CXCL14 in the central nervous system could be a potential target for anti-hyperphagic therapy. We are making efforts to identify CXCL14 receptors and to establish sensitive bioassay systems for future therapeutic applications.

## Materials and Methods

### Animal experiments

*CXCL14* ^+/−^ mice were backcrossed with C57BL/6 mice (Nihon SLC, Hamamatsu, Japan) for more than 10 generations and intercrossed with each other to obtain *CXCL14* ^+/+^, *CXCL14* ^+/−^ and *CXCL14* ^−/−^ littermates. For some experiments, *CXCL14* ^−/−^ male and *CXCL14* ^+/−^ female mice were crossed to produce *CXCL14* ^+/−^ and *CXCL14* ^−/−^ mice. Mice were fed a standard diet (CE-2) (Nihon CLEA, Tokyo, Japan). KKA^y^ (mixed background) and *ob*/+ mice (C57BL/6 background) were purchased from Nihon CLEA and Charles River (Yokohama, Japan), respectively. A^y^ mice (C57BL/6 background) were imported from The Jackson Laboratory (Bar Harbor, ME). All mice were maintained under a 12-hour light, 12-hour dark cycle in a pathogen-free animal facility. All experimental procedures involving mice were pre-approved by Ethical committee of Animal Experiments in The Tokyo Metropolitan Institute of Medical Science, and performed according to the guidelines for Proper Conduct of Animal Experiments (http://www.scj.go/en/animal/index.html).

### Metabolic measurements

Serum concentrations of growth hormone and IGF-I were determined using ELISA kits from Shibayagi (Shibukawa, Gumma, Japan) and R&D Systems (Minneapolis, MN), respectively. Mouse core body temperature was measured using an electric thermometer with a probe (Muromachi Kikai, Tokyo, Japan).

### Oxygen consumption and locomotor activity

Oxygen consumption of female mice was measured using an O_2_/CO_2_ metabolism measurement system (MK-5000, Muromachi Kikai) under the fasting condition. Spontaneous locomotor activity and food intake of mice were recorded using a laboratory animal monitoring system (ACTIMO/MFD-100, Shinfactory, Fukuoka, Japan).

### Quantitative RT-PCR

Total RNA was reverse-transcribed using High Capacity RNA-to-cDNA Master Mix (Applied Biosystems, Foster City, CA). Real-time PCR was performed with SYBR premix Ex Taq II (Takara, Otsu, Japan) using a LightCycler480 system (Roche Applied Science, Indianapolis, IN). Messenger RNA expression levels were determined using Relative Quantification Software with *Glyceraldehyde 3-phosphate dehydrogenase* (*GAPDH*) as an internal control. Primer sequences are listed in Table 2.

### ICV and IP injection

For ICV injection, female mice were anesthetized with xylazine (10 mg/Kg) plus ketamine (100 mg/Kg) and placed in a Kopf stereotaxic frame (Koft Instruments, Tujunga, CA). Then a chronic double-walled stainless steel cannula was stereotaxically implanted into the lateral ventricle of each mouse according to the atlas book [G. Paxinos & K. B. J. Franklin: The Mouse Brain *in Stereotaxic Coordinates*, Second edition (Academic Press, Inc., San Diego, 1997)]. The stereotaxic coordinates for the lateral ventricle were AP 0.2 (0.2 mm posterior to bregma), L 1 (1 mm left from mid-sagittal line) and H 2.4 (2.4 mm below bregma). Two weeks after the surgery, unanesthetized mice were injected with 2 µl (2 µg) of 100 nM human CXCL14 (PeproTech, Rocky Hill, NJ) in PBS or PBS over 1 minute via an inner cannula using a Hamilton syringe. ICV injections were performed less than 1 hour before beginning of the dark period. All mice were handled daily to habituate them to experimental maneuvers.

IP injection was carried out without anesthesia just before beginning of the dark period. After two-day adaptation with PBS injections, mice were intraperitoneally injected with 200 µl of human CXCL14 in PBS at 2 µg/g body weight or PBS with a 26G syringe. Serum concentrations of CXCL14 in mice were determined by using DY866 DuoSet ELISA kit (R&D Systems).

### Statistical analysis

All statistical analysis was performed using ANOVA repeated measures analysis (Statview J5.0, Abacus Concepts). Between factors related to the breeding and feeding conditions in home cages were regarded to be identical among different mouse strains. A *P*-value of <0.05 was considered significant for the unpaired Student's *t*-test.
